# Relevance of anatomy to medical education and clinical practice: perspectives of medical students, clinicians, and educators

**DOI:** 10.1007/s40037-016-0310-4

**Published:** 2016-10-26

**Authors:** Amgad Sbayeh, Mohammad A. Qaedi Choo, Kathleen A. Quane, Paul Finucane, Deirdre McGrath, Siun O’Flynn, Siobhain M. O’Mahony, Colm M. P. O’Tuathaigh

**Affiliations:** 1 Department of Anatomy and Neuroscience, School of Medicine, University College Cork, Cork, Ireland; 2 Medical Education Unit, School of Medicine, University College Cork, Cork, Ireland; 3 Graduate Entry Medical School, University of Limerick, Limerick, Ireland

**Keywords:** Medical education, Anatomy, Curriculum development, Mode of entry to medical school

## Abstract

**Introduction:**

Against a backdrop of ever-changing diagnostic and treatment modalities, stakeholder perceptions (medical students, clinicians, anatomy educators) are crucial for the design of an anatomy curriculum which fulfils the criteria required for safe medical practice. This study compared perceptions of students, practising clinicians, and anatomy educators with respect to the relevance of anatomy education to medicine.

**Methods:**

A quantitative survey was administered to undergraduate entry (*n* = 352) and graduate entry students (*n* = 219) at two Irish medical schools, recently graduated Irish clinicians (*n* = 146), and anatomy educators based in Irish and British medical schools (*n* = 30). Areas addressed included the association of anatomy with medical education and clinical practice, mode of instruction, and curriculum duration.

**Results:**

Graduate-entry students were less likely to associate anatomy with the development of professionalism, teamwork skills, or improved awareness of ethics in medicine. Clinicians highlighted the challenge of tailoring anatomy education to increase student readiness to function effectively in a clinical role. Anatomy educators indicated dissatisfaction with the time available for anatomy within medical curricula, and were equivocal about whether curriculum content should be responsive to societal feedback.

**Conclusions:**

The group differences identified in the current study highlight areas and requirements which medical education curriculum developers should be sensitive to when designing anatomy courses.

## What this paper adds

- Students, clinicians, and anatomy educators differ with respect to perceptions of the relevance of anatomy education to medicine and clinical practice.
- Collection of data related to current trends in the content and mode of delivery of anatomy curricula provides an evidence base for evaluating whether existing curricula meet the needs of the modern medical workforce.
- Comparison of student, educator, and clinicians perspectives highlights the challenge of tailoring anatomy education to both foster the development of professionalism, and to increase student readiness to function effectively in a clinical role.

## Introduction

Recent publications have shown that modern medical curricula significantly undervalue essential anatomy teaching in terms of student contact hours [1, 2]. In response to decreased contact hours, it has been suggested that anatomy education should focus more closely on a subset of the most clinically relevant topics [3, 4]. Relevant to this point is a recent survey of 93 physicians across US medical schools, who were asked to assess the relative importance of various topics taught on a human gross anatomy course [5]. They reported that the perceived importance of specific anatomical topics was largely determined by the respondent’s area of speciality and/or clinical affiliation. However, medical imaging was ranked as both highly important and clinically relevant by all respondents.

Although graduate entry to medicine (GEM) has been the only mode of medical school entry in North America for many years, it is a recent development in Europe where the majority of medical students commence their studies in medicine after completion of secondary level education (undergraduate/direct-entry medicine; DEM). GEM programmes were originally developed to increase diversity in the medical workforce [6, 7], with a view to training doctors from more diverse socioeconomic and academic backgrounds [8]. Significant differences across motivational variables as well as approaches to learning have been identified between DEM and GEM students (e. g. Sandover et al.) [9]. As the nature of medical training and academic clinical training pathways continue to evolve, it is important that the learning requirements and educational preferences of graduate entrants are also evaluated.

The collection and analysis of data related to current trends in the content and mode of delivery of anatomy curricula is expected to provide an evidence base for assessing whether existing curricula meet the needs of the modern medical workforce. To that end, a survey was designed and tailored for medical students, clinicians, and academic anatomy educators to: (1) investigate perceptions of the relevance of anatomy curricula to the modern medical curriculum as well as current medical practice based on the following survey domains: (a) level of agreement, or otherwise, with statements concerning the role of anatomy in the medical curriculum and its relevance to clinical practice; (b) level of satisfaction with time allocated for anatomy within curriculum; (c) ranking, in terms of perceived importance, of various modes of instruction in anatomy education. Based on previous survey-based studies, it was hypothesized that students’ perspectives of the role of human anatomy in the medical curriculum would differ from anatomy educators, and that both viewpoints would differ again from that of clinical practitioners [5, 10]; (2) investigate differences in perceptions of anatomy education among current medical students based on mode of entry to medical school (i. e. GEM *vs*. DEM). In light of research indicating qualitative differences in motivational and cognitive factors between GEM and DEM students, the hypothesis was that the responses of both student groups would diverge across both survey domains.

## Methods

The present study employed a cross-sectional, questionnaire-based design. This study was carried out in the School of Medicine at University College Cork (UCC), Cork, Ireland, and Graduate Entry Medical School, University of Limerick (UL), Limerick, Ireland. Questionnaire data was collected between August 2012 and April 2013. Ethical approval was obtained from the School of Medicine Research Ethics Committee and the Research Ethics Committee of the Cork Teaching Hospitals (ECM 4(r) 07/08/2012), as well as the University Faculty Research Ethics Committee at the University of Limerick, Limerick, Ireland.

The study sample comprised: (a) medical undergraduates enrolled in the DEM and GEM programmes at UCC; (b) students enrolled in the 4‑year GEM programme at UL; (c) clinicians who had graduated from UCC between 2005 and 2013; (d) university-based educators involved in the delivery of undergraduate anatomy courses to medical students in British and Irish medical schools. For both UCC DEM and GEM students, anatomy content is delivered *via* lectures and cadaver-based teaching, using prosection- and dissection-based activities, together with availability of medical imaging and multimedia resources. In contrast, anatomy in the UL GEM programme is taught through non-cadaveric models and computer-assisted learning.

A quantitative survey was developed for each of the three study populations (DEM/GEM students, clinicians, anatomy educators), where items related to relevance of anatomy to medical education were based directly on World Federation for Medical Education quality standards for integrating basic sciences into medical curricula [11]. All questions related to curriculum duration and instructional methods were developed following detailed consultation involving faculty and staff involved in anatomy education at UCC. A panel of three anatomy educators provided ratings regarding whether each item was essential and a content validity ratio [12] of 1.0 was a pre-requisite for inclusion in this study. Each version of the survey tool assessed four domains: (a) demographics and educational background; (b) relationship between anatomy and medical education and clinical practice; (c) curriculum duration; (d) mode of instruction. The majority of the survey items employed a Likert scale response format, where the respondent was presented with a series of statements and was required to express agreement or indicate importance using a five-point scale (e. g. 1 = strongly disagree, 2 = disagree, 3 = neutral, 4 = agree, and 5 = strongly agree). The remaining items consisted of ranking, binary- or multiple-choice response format items.

All data are summarized as percentages or mean values (±standard error of the mean, SEM) and are illustrated in tables. To examine differences between student groups’ (UCC DEM *vs*. UCC GEM *vs*. UL GEM) Likert scale responses to statements describing the role of anatomy in medical education, independent t‑test comparisons were conducted. To control for familywise error rate across the planned comparisons (36 tests in total), a Bonferroni adjusted alpha level of 0.0015 per test was employed. Where respondents were asked to state level of agreement with statements describing the role of anatomy in medical education, the wording of the items varied significantly depending on group membership (students, clinicians, anatomy educators), thereby precluding any statistical comparisons across these three groups. The only exception was the statement ‘Anatomy is/was relevant to my education in medical school’, which was worded similarly for medical students and clinicians. One-way analysis of variance (ANOVA) was employed to test differences between all groups (UCC DEM vs. UCC GEM vs. vs. UL GEM vs. clinicians vs. anatomy educators) in relation to ranking of anatomy teaching methods, and the same test was used to compare students’ and clinicians’ responses to the statement ‘Anatomy was relevant to my education in medical school’. The threshold for statistical significance was set at *p* < 0.05. Where appropriate, *post-hoc* comparisons were carried out using independent t‑tests. To control for familywise error rate across post-hoc comparisons (90 tests in total), a Bonferroni adjusted alpha level of 0.0006 per test was employed. Pearson’s chi square analysis was used to examine the association between student group membership (UCC DEM vs. UCC GEM vs. UL GEM) and all other items requiring a categorical response. The threshold for statistical significance was set at 0.05. Statistical analyses were carried out using IBM SPSS (v. 20; IBM SPSS, Armonk, NY).

## Results

In total, 484 (out of a total eligible population of 749) UCC students from both the DEM and GEM programmes completed the survey, indicating a response rate of 64.6 %. The number of UCC DEM survey respondents was 352 (72.3 %), while the total number of GEM respondents was 132 (27.7 %). Eighty-seven GEM students from UL responded, yielding a response rate of 20.0 % (87/436).

Altogether 146 clinicians (out of an estimated total of 1034 eligible participants) completed the survey, yielding a response rate of 14.1 %. This response rate is comparable with that reported for other online surveys conducted in epidemiological studies [13]. Of the 89 academic staff members contacted at British and Irish medical schools *via* web-based survey invitation, 30 completed the questionnaire, yielding a response rate of 33.7 %.

The mean age of study participants was as follows (mean ± SEM): UCC DEM (20.7 ± 0.8), UCC GEM (27 ± 1.0), UL GEM (28.3 ± 2.1), clinicians (28 ± 1.8), anatomy educators (44.6 ± 3.1). The gender distribution (% of males) within the sample was as follows: UCC DEM (41), UCC GEM (56), UL GEM (40), clinicians (43), and anatomy educators (63).

### Relationship between anatomy curriculum and medical education

Table 1 describes agreement ratings for statements addressing the role of anatomy within the undergraduate medical curriculum in UCC DEM, UCC GEM, and UL GEM students.

**Table 1:** Mean (±SEM) agreement ratings (on a 1–5 Likert scale, where 1 = ‘Strongly Disagree’ and 5 = ‘Strongly Agree’) for statements related to linkage between anatomy education and medical education and clinical practice, in UCC direct-entry medicine (DEM; *n* = 352), UCC graduate-entry medicine (GEM; *n* = 132), and UL GEM students (*n* = 87). Independent t‑test comparisons, where *p* < 0.0015 (two-tailed) (Bonferroni correction)

	UCC DEM (*n* = 352)	UCC GEM (*n* = 132)	UL GEM (*n* = 87)
	Mean ranking (±SEM)	Mean ranking (±SEM)	Mean ranking (±SEM)
Anatomy is relevant for my education at medical school	4.8 (0.02)	4.8 (0.04)	4.9 (0.06)
Patient/cadaver contact is important towards acquiring sufficient clinical knowledge and skills	4.6 (0.03)^a^	4.5 (0.07)^b^	3.6 (0.14)
Anatomy is important for professional development	4.4 (0.04)^a^	4.4 (0.06)	4.1 (0.11)
Anatomy has helped me to link basic and clinical sciences	4.2 (0.04)	4.1 (0.05)	4.1 (0.09)
I am able to understand the structure of the human body through anatomy education	4.5 (0.03)^a,c^	4.3 (0.05)^b^	3.5 (0.13)
Anatomy education is important for lifelong, self-directed learning	3.8 (0.04)^a,c^	3.6 (0.06)^b^	4.2 (0.10)
I understand the link between anatomy education and postgraduate training or clinical practice	3.9 (0.04)	3.9 (0.06)	3.9 (0.09)
Anatomy education has helped me to understand diagnostic imaging	3.7 (0.05)	3.6 (0.08)^b^	3.9 (0.11)
Anatomy education has contributed to development of professionalism skills	3.7 (0.05)^a,c^	3.4 (0.08)^b^	3.1 (0.11)
Anatomy has improved my understanding of the principles of scientific method and evidence-based medicine	3.5 (0.08)^c^	3.1 (0.09)	3.5 (0.12)
Anatomy education has improved my teamwork and communication skills	3.6 (0.05)^a,c^	3.2 (0.07)	3.2 (0.11)
Anatomy education has helped me to develop awareness of ethics in medicine	3.4 (0.05)^a,c^	3.2 (0.08)^b^	2.7 (0.10)

^a^UCC DEM significantly different to UL GEM; ^b^UCC DEM significantly different to UCC GEM; ^c^UCC GEM significantly different to UL GEM

As indicated in Table 1, UCC DEM provided higher agreement ratings relative to UCC GEM and/or UL GEM for the following areas of relevance: anatomy and patient/cadaver contact (UL GEM, t (436) = 9.84, *p* < 0.0001); anatomy and structure of human body (UCC GEM, t (482) = 3.30, *p* = 0.001; UL GEM, t (436) = 10.64, *p* < 0.0001); anatomy and professionalism (UCC GEM, t (481) = 3.30, *p* = 0.001; UL GEM, t (435) = 5.61, *p* < 0.0001); anatomy and ethics of medicine (UCC GEM, t (480) = 3.15, *p* = 0.001; UL GEM, t (437) = 6.97, *p* < 0.0001); anatomy and teamwork and communication skills (UCC GEM, t (481) = 4.64, *p* < 0.0001; UL GEM, t (437) = 4.29, *p* < 0.0001). UL GEM demonstrated higher agreement ratings relative to UCC DEM for the following area: anatomy and self-directed learning (t (437) = 4.00, *p* = 0.001).

UCC GEM demonstrated greater agreement ratings relative to UL GEM for the following statements: anatomy and patient/cadaver contact (t (216) = 6.40, *p* < 0.0001); anatomy and structure of human body (t (216) = 6.03, *p* < 0.0001); anatomy and ethics of medicine (t (215) = 3.79, *p* < 0.0001).

Table 2 summarizes mean agreement ratings (±SEM) for statements addressing the role of anatomy in the undergraduate medical curriculum and its relationship with clinical practice in clinicians and anatomy educators, respectively. Clinicians de-emphasized the link between anatomy education and professionalism, principles of scientific method and evidence-based medicine, and awareness of ethics of medicine (agreement rating range = 3.2–3.5), while also minimizing its contribution to lifelong, self-directed learning, teamwork and communication skills, and critical thinking. Anatomy educators provided generally high agreement ratings for each of the statements; the statement which received least support indicated that educators were equivocal about whether the course should be responsive to feedback from the wider society.

**Table 2:** Mean (±SEM) agreement ratings (on a 1–5 Likert scale, where 1 = ‘Strongly Disagree’ and 5 = ‘Strongly Agree’) for statements related to linkage between anatomy education and medical education and clinical practice in clinicians and anatomy educators

Statements	Mean agreement rating [1–5]	SEM
*Clinicians*		
Anatomy was relevant to my education in medical school	4.5	0.09
I understand the link between anatomy education and my current clinical practice role	4.1	0.12
As part of the anatomy curriculum, patient/cadaver contact is important towards acquiring sufficient clinical knowledge and skills	4.1	0.12
Anatomy education has helped me to understand diagnostic imaging and how to interpret various imaging scans	4.1	0.13
Anatomy education played an important role in my professional development	3.9	0.16
Anatomy has helped me to link my knowledge of basic sciences with clinical sciences	3.8	0.11
Anatomy education contributed to my readiness to function effectively in my current clinical role	3.7	0.15
Anatomy education improved my teamwork and communication skills	3.5	0.13
Anatomy education helped me to develop my awareness of the ethics of medicine	3.5	0.15
Anatomy education contributed to the development of my professionalism skills	3.4	0.15
Anatomy has improved my understanding of the principles of scientific method and evidence-based medicine, including analytical and critical thinking	3.2	0.14
Teaching methods used to teach anatomy in my university helped prepare students for lifelong, self-directed learning	3.1	0.13
*Anatomy educators*		
Anatomy education plays a crucial role in the integration of basic sciences and clinical sciences	4.6	0.10
Medical imaging is an important pedagogical tool in medical education	4.4	0.09
The anatomy curriculum should contribute to the development of understanding of the scientific knowledge, concepts and methods fundamental to acquiring and applying clinical science	4.3	0.11
Anatomy education contributes to the development of professionalism skills in medical students	4.3	0.13
The anatomy curriculum should ensure students have sufficient patient/cadaver contact, in order to acquire sufficient clinical knowledge and skills to have appropriate clinical responsibility	4.3	0.15
Anatomy education is important in the development of awareness of the ethics of medicine	4.2	0.14
The anatomy curriculum should seek input from the environment in which medical graduates will work	4.1	0.11
Instructional methods used to teach anatomy in my university helped prepare students for lifelong, self-directed learning	4.0	0.17
Anatomy education within the medical curriculum should include elements for training students in scientific thinking and research methods	3.9	0.16
Computerised learning and multimedia packages will play an increasingly important role in anatomy education	3.8	0.12
The anatomy curriculum should be expected to undertake course modification in response to feedback from the wider community and society	3.5	0.14

Univariate comparison of agreement ratings for the statement ‘anatomy is/was relevant to my education in medical school’ across the clinician and student groups (DEM, GEM) revealed no significant group difference (F (1, 482) = 2.21, *p* > 0.05).

### Curriculum duration

When asked whether the time dedicated to anatomy education within the curriculum was ‘too little’, ‘too much’, or ‘sufficient’, UCC DEM were most likely to indicate that it was ‘sufficient’ (89 %), followed by UCC GEM (79 %) and UL GEM students (48 %) (χ_2_ = 11.20, *p* < 0.004). Among clinicians, 80 % indicated that the time dedicated to anatomy was ‘sufficient’, while 9 % indicated that it was ‘too little’. Of the anatomy educators, 50 % felt that there was ‘too little’ time dedicated to anatomy, with remaining educators satisfied that it was sufficient.

### Mode of anatomy instruction

Table 3 provides a summary of mean rankings (±standard error of the mean, SEM), based on perceived importance, for anatomical teaching methods in UCC DEM, UCC GEM, UL GEM, clinicians, and anatomy educators and a description of results of ANOVA analyses and subsequent *post-hoc* group comparisons in relation to ranking of teaching methods.

**Table 3:** Mean (±SEM) ranking of anatomical teaching methods in UCC direct-entry medicine (DEM; *n* = 352), UCC graduate-entry medicine (GEM; *n* = 132), UL GEM (*n* = 87), clinicians (*n* = 146), anatomy educators (*n* = 30), based on importance where 1 = most important and 10 = least important

	UCC DEM (*n* = 352)	UCC GEM (*n* = 132)	UL GEM (*n* = 87)	Clinicians (*n* = 146)	Anatomy Educators (*n* = 30)	*P*-value*
	Mean ranking (±SEM)	Mean ranking (±SEM)	Mean ranking (±SEM)	Mean ranking (±SEM)	Mean ranking (±SEM)	
Lectures	3.1 (0.01)^b^	3.1 (0.03)	4.6 (0.03)	2.7 (0.01) ^b^	3.7 (0.08)	0.0001
Prosection	2.4 (0.01)^a, b, c^	2.2 (0.02)^d, e^	6.5 (0.04)	3.9 (0.02) ^b^	4.1 (0.08)^b^	0.0001
Dissection	4.1 (0.01)	4.0 (0.04)	4.9 (0.04)	2.9 (0.01) ^b^	3.5 (0.09)	0.0001
CAL	5.9 (0.02)	5.1 (0.03)	6.2 (0.02)	4.9 (0.02) ^b^	6.3 (0.08)	0.0001
Small-group learning	5.4 (0.01)	5.5 (0.03)	5.2 (0.03)	4.5 (0.01)	4.5 (0.07)	0.001
Demonstrator-lead SGL	3.4 (0.01)	3.8 (0.03)	3.7 (0.03)	3.9 (0.02)	4.0 (0.08)	0.234
Formative assessments	4.8 (0.01)^b^	4.8 (0.03)	5.8 (0.02)	6.3 (0.01)	6.1 (0.05)	0.0001
Self-directed learning	7.1 (0.01)^a^	6.3 (0.03)^d^	5.0 (0.03)^ d^	5.4 (0.02)	6.5 (0.07)	0.0001
Case-based sessions	7.7 (0.01)	6.7 (0.02)^d^	4.5 (0.03)^ b, d^	6.2 (0.03)	7.6 (0.08)	0.0001
Other	9.1 (0.01)	8.2 (0.02)^d^	9.0 (0.02)	9.3 (0.01)	8.6 (0.06)	0.002

*CAL* computer-assisted learning, *SGL* small-group learning

*p < 0.05 (ANOVA comparisons). Post-hoc t‑test comparisons, where *p* < 0.0006 (two-tailed) (Bonferroni correction)

^a^significantly higher than UCC GEM, ^b^significantly higher than UL GEM, ^c^significantly higher than medical educators, ^d^significantly higher than UCC DEM, ^e^significantly higher than clinicians

## Discussion

This cross-sectional study is the first of its kind to compare the different perspectives of medical students, clinicians, and anatomy educators regarding the relevance and relationship between anatomy education and the educational outcomes associated with the contemporary medical curriculum. Previous studies have described these perspectives in either one group only, e. g. clinicians, medical students, or educators (see Finucane et al. and Larzarus et al. for review of these findings) [8, 10]. Alternatively, they have compared different views of students, clinicians, and/or educators but have focused only on the perceived clinical relevance and applicability of specific anatomy course content [10]. In agreement with previous studies, which have shown differences in both motivational variables and approaches to learning in GEM *vs*. DEM (e. g. Sandover et al) [9], this study has also demonstrated differences between both student groups in relation to preferred mode of instruction and perceived relationship between anatomy and clinical education.

### Role of anatomy in the medicawl curriculum and its relevance to clinical practice

All groups showed a high level of agreement with the proposition that anatomy education was an important element of the medical curriculum. However, the agreement rating data indicated that clinicians were equivocal about whether anatomy education contributed to their ability to function effectively in a clinical role. This may also underlie their perception that anatomy education has not contributed majorly to professionalism-related competencies. Clinicians showed comparatively low agreement ratings (ranging from 3.1 to 3.4 on the 5‑point scale) with statements indicating that anatomy promoted professionalism, teamwork and communication skills, as well as critical thinking and scientific methods. It has been suggested that the anatomy course initiatives including the introduction of inter-professional activities at the preclinical level (including anatomy) may help to foster a sense of professional identity, and increase competencies in areas such as teamwork, communication, and awareness of ethics [5]. However, a recent intervention-based study, which aimed to integrate professionalism into anatomy teaching using a case-study-discussion intervention, reported that the intervention was largely without effect on various outcome measures of professionalism [14]. The present study highlights the perceived lack of relevance of anatomy education to such competencies among selected groups (i. e. clinicians, as well as GEM students where the same pattern was observed), and is suggestive of a complex relationship between anatomy course content and teaching of medical professionalism. Specifically, it is proposed that the present data might reflect the tendency for medical students and recently graduated clinicians to view professionalism as a topic which can only be addressed in a clinical setting, and ideally based on observation of doctors rather than formal teaching [15]. However, recent research indicates that relatively junior clinicians in the present study may not fully appreciate the relevance of anatomy content to diagnostic reasoning and clinical decision-making. It has been shown that junior medical doctors in particular use anatomical knowledge in all phases of the consultation, especially during physical examination [16].

It is generally recognized that the viewpoint of clinicians should be taken into account if the aim is to develop an anatomy curriculum relevant to daily clinical practice [10]. Previous work has shown that while there may be areas of clinical care which rely to a greater (e. g. imaging and diagnostics) or lesser (e. g. doctor-patient communication) extent on anatomical knowledge, all groups in the current study (echoing previously published results) rated clinical relevance as a crucially important element of the anatomy curriculum.

While all participants were in agreement that patient/cadaver contact is important, it was clear that GEM students (in particular UL GEM students) found this to be less of a priority, reflecting the non-cadaveric nature of the UL GEM programme which focuses on diagnostic imaging and case-based problem solving. All agreed that anatomy teaching is important for integrating basic and clinical science content. It has been found that the introduction of a greater clinical focus in basic science teaching may help to bridge the gap between basic science and clinical practice [17]. All participants in this study agreed with the statement that anatomy education is closely related to reading of diagnostic imaging scans. The clinical images may help students to put into perspective the significance of learning the anatomical information and allow them to apply their newly learned anatomical knowledge to real-life situations. Anatomy teaching programmes incorporating radiological images tools such as MRI, X‑ray, CT, have generally proven successful [18].

### Anatomy in the medical curriculum and its relevance to clinical practice: GEM *vs*. DEM

GEM students were also significantly less likely than their DEM colleagues to agree that anatomy education contributed to the development of professionalism or their awareness of ethics in medicine, or that it improved their teamwork and communication skills. It has been suggested that international GEM curricula often seek to harness their broader life and work experience, allowing them to develop the more equal partnerships, encouraging reflective practice, and more outward looking perspectives that underlie medical professionalism [19]. One explanation for the perceived lack of focus on professionalism within the anatomy curriculum among GEM students is that it reflects the increased academic load which is a consequence of the compression of the traditional five-year courses into the four-year GEM timeframe. Several initiatives designed to foster professionalism development within the anatomy curriculum, including meeting the families of anatomical donors (e. g. Halliday et al) [20], can be logistically challenging to incorporate into crowded and time-poor GEM curricula. Another explanation is that it might reflect differences between GEM and DEM students with respect to attitudes to professional behaviours and medical professionalism [21]. When medical undergraduates (particularly GEM) were asked how they felt professionalism is best taught, role models and learning through experience were identified as being the most useful instructional techniques [21]. These results would suggest that GEM students in the present sample find it more difficult than their DEM counterparts to appreciate the relationship between anatomy curricular content and professionalism, well as transferable skills including teamwork and critical thinking.

### Time allocated for anatomy within the medical curriculum: educator perspectives

Educators expressed dissatisfaction with the number of hours dedicated to anatomy teaching within the curriculum; this sentiment is congruent with reports of increasingly fewer hours dedicated to anatomy education within medical curricula [1, 3]. Additionally, educators also displayed little enthusiasm for the proposal that they should undertake course modification in response to feedback from the wider community and society.

### Mode of anatomy instruction

Lecturing and anatomical laboratory practicals are the main preferred methods for learning and teaching anatomy across all groups. These findings also support the use of cadavers as a tool for teaching by either dissection or prosection. Some studies have shown that dissection is essential for understanding and learning anatomy, and that retention is highest when learning is combined with experience of dissection [17]. It has been suggested that active observation and participation in cadaveric dissection guided by demonstrators helps the understanding of three-dimensional structures and enhances development and attitudes towards teamwork [17].

Demonstrator-led small group teaching was also rated by all stakeholders as one of the three most important teaching methods. A recent comparison of structured vs*.* unstructured anatomy learning environments revealed that levels of anatomical knowledge for the strictly guided, station-based learning group were higher than for the loosely guided, self-directed learning group. These data suggest that unstructured learning environments may be less appropriate for junior learners, who may lack an appropriate mental framework and are therefore hindered in taking up new information [22]. In this context, it is noteworthy that the preference for demonstrator-led teaching was highest among the youngest students cohort, the DEM cohort, in the present study.

One important question arising from these data is whether medical education curriculum developers should in fact take into account research evidence for preferred pedagogical methodologies and/or individual learning strategies and requirements of specific student cohorts when designing anatomy courses. Kirschner and Van Merrienboer [23] suggested that one of the most popular ‘urban legends’ in education is the pervasive notion that learners know best how to adapt learning to their own preferred learning styles. The assumption arising from this notion is that teachers or instructional material developers should take into account their preferences so as to facilitate their learning and help them achieve the best possible learning outcomes. However, it is known that preference for a particular mode of instruction is not correlated with improved productivity among those who experience their preferred instructional technique [23]. Others have suggested that self-regulated learning theory should be invoked to explain the relationship between students’ learning engagement and academic performance in anatomy courses [24]. Self-regulated learning theory focuses on the active participation of students in personal, behavioural, motivational, and cognitive efforts to achieve valued academic goals. In this context, level of self-regulation modulates students’ employment of learning strategies, engagement with anatomy course material, and the extent to which activities and taught sessions are linked with expected learning outcomes [24]. Therefore, a failure of self-regulation might underlie the reported disconnect between preferred teaching methods and academic achievement. With respect to implications for course development, rather than focus on tailoring the curriculum to match the stated preferences of specific learner cohorts, it has been proposed that it may be more valuable to focus on the fundamental things that learners have in common rather than on the multitude of styles on which they may be different from each other [23].

### Limitations

One of the limitations of the present study was the limited sample size achieved for anatomy educators, and the likely diversity within the entire sample with respect to experience and familiarity with various anatomy teaching methods. Additionally, among the clinicians, several of the teaching methods referred to in the survey, including computer-assisted learning, would not be as easily available as they are for current medical students. In a similar vein, while differences in how anatomy is taught between UCC and UL graduate-entry programmes produced a number of interesting differences in perceptions and attitudes between both groups, their different experiences of anatomy education provides a degree of context to the observed differences in attitudes/perceptions. Another limitation of the present study is that selected types of validity (e. g. predictive, construct) and reliability were not established for the survey tool employed in this study. Measurement of validity was limited to contact validity, bearing in mind that many of the survey items were based on content adapted from the World Federation for Medical Education guidelines for integrating basic sciences into medical curricula [25]. Lastly, the response rate for the clinician sample (14.1 %) is considerably lower than that reported for the other groups in the current study. This is likely due to a combination of factors including a busy clinical schedule, inaccurate mailing list information, and the difficulty associated with increase in web-based survey requests in recent years.

## Conclusions

Each of the groups (students, clinicians, and anatomy educators) in the present study expressed different levels of agreement/disagreement with statements describing the linkage between anatomy teaching and medical education and clinical practice. Clinicians were more likely to question the link between anatomy education and clinical practice, suggesting that anatomy teaching should be more closely integrated with clinical teaching and exposure during the later years of the programme. GEM students across both institutions polled in the present study (UCC and UL), and clinicians, were found to be less likely than DEM students to agree that anatomy education contributed to the development of professionalism-related competencies. The study reported several group differences with respect to preferred instructional methods, with lectures and laboratory practicals still rated highly across all groups. However, despite the observed group differences, it is suggested that curriculum designers’ selection of instructional methods should be sensitive to common preferences across learners or students cohorts rather than seeking to tailor mode of instruction to individual or group differences.
